# Laboratory Diagnosis of Paratyphoid Fever: Opportunity of Surface Plasmon Resonance

**DOI:** 10.3390/diagnostics10070438

**Published:** 2020-06-28

**Authors:** Dina M. Alhaj-Qasem, Mohammad A. I. Al-Hatamleh, Ahmad Adebayo Irekeola, Muhammad Fazli Khalid, Rohimah Mohamud, Aziah Ismail, Fatin Hamimi Mustafa

**Affiliations:** 1 Precision Medical Laboratory, Zahran, Amman 11183, Jordan; dina.dq96@gmail.com; 2 Department of Immunology, School of Medical Sciences, Universiti Sains Malaysia, Kubang Kerian, Kelantan 16150, Malaysia; alhatamleh@student.usm.my (M.A.I.A.-H.); rohimahm@usm.my (R.M.); 3 Department of Medical Microbiology and Parasitology, School of Medical Sciences, Universiti Sains Malaysia, Kubang Kerian, Kelantan 16150, Malaysia; profahmad007@yahoo.com; 4 Microbiology Unit, Department of Biological Sciences, College of Natural and Applied Sciences, Summit University Offa, Offa PMB 4412, Kwara State, Nigeria; 5 Institute for Research in Molecular Medicine (INFORMM), Universiti Sains Malaysia, Kubang Kerian, Kelantan 16150, Malaysia; fazlikhalid@usm.my (M.F.K.); aziahismail@usm.my (A.I.); 6 Hospital Universiti Sains Malaysia, Kubang Kerian, Kelantan 16150, Malaysia

**Keywords:** *Salmonella* Paratyphi, paratyphoid fever, bacterial detection, SPR, optical sensor, biosensor

## Abstract

Paratyphoid fever is caused by the bacterium *Salmonella* *enterica* serovar Paratyphi (A, B and C), and contributes significantly to global disease burden. One of the major challenges in the diagnosis of paratyphoid fever is the lack of a proper gold standard. Given the absence of a licensed vaccine against *S.* Paratyphi, this diagnostic gap leads to inappropriate antibiotics use, thus, enhancing antimicrobial resistance. In addition, the symptoms of paratyphoid overlap with other infections, including the closely related typhoid fever. Since the development and utilization of a standard, sensitive, and accurate diagnostic method is essential in controlling any disease, this review discusses a new promising approach to aid the diagnosis of paratyphoid fever. This advocated approach is based on the use of surface plasmon resonance (SPR) biosensor and DNA probes to detect specific nucleic acid sequences of *S.* Paratyphi. We believe that this SPR-based genoassay can be a potent alternative to the current conventional diagnostic methods, and could become a rapid diagnostic tool for paratyphoid fever.

## 1. Introduction

The genus *Salmonella*, which belongs to the *Enterobacteriaceae* family, consists of rod-shaped Gram-negative facultatively anaerobic bacteria [1]. It includes various types and subtypes, according to the ever-changing and improving classification systems. Initially, the types of *Salmonella* genus were divided based on epidemiology and the clinical syndromes they cause, and every subtype was considered a species [2]. Antigenic serotypes detailed in the Kauffman-White scheme provides more accurate classification by designating each subset of the bacteria with a unique variant of O (somatic, a lipopolysaccharide of the outer membrane) and H (flagellar) antigens, yielding about 2600 serovars of *Salmonella* [3]. However, fewer than 100 serovars are known to be harmful to humans and are well-studied, while others have rarely been extensively examined [4].

Recent methods to classify the *Salmonella* genus are founded on genetic studies of the bacteria. Over the years, the *Salmonella* genus, with its near 2600 serovars, was divided into two main *Salmonella* species; *S. enterica* and *S. bongori* [5]. More recently, a new species, *S. subterranean*, which showed 96.4% similarity to *S. bongori* was reported [6]. The two main species have also been subdivided into subspecies. The *S. enterica* species involves subspecies *enterica* (subspecies I) that includes 1531 serovars, subspecies *salamae* (subspecies II—505 serovars), subspecies *arizonae* (subspecies IIIa—99 serovars), subspecies *diarizonae* (subspecies IIIb—336 serovars), subspecies *houtenae* (subspecies IV—73 serovars), and subspecies *indica* (subspecies VI—13 serovars) [3]. The *S. bongori* species, on the other hand, was formerly considered subspecies V, but later distanced as a separate species that includes 22 serovars [3,7]. The main subspecies infectious to humans is subspecies I (*enterica*) [8]. Depending on clinical syndromes they cause in humans, *Salmonella* can be categorized into two types; typhoidal and nontyphoidal *Salmonella* [9]. The former contains serotypes that can cause enteric fever, while nontyphoidal *Salmonella* includes all other serotypes. In addition, the main serotypes that cause enteric fever have been classified into two types; *S.* Typhi and *S.* Paratyphi (which includes *S.* Paratyphi A, B, and C) [10].

Although typhoid and paratyphoid fever are caused by different types of *S. enterica* (*S.* Typhi and *S.* Paratyphi), they are, to some extent, considered to cause a single disease (i.e., enteric fever). Studies have shown that it is impossible to differentiate clinically between paratyphoid and typhoid fever [11], thus, the diagnosis and management of paratyphoid cases have mainly relied on experience gained from typhoid reports [12,13]. Reports on the paratyphoid fever are still contradictory [12,13]. One major problem within resource-limited areas where standard laboratory approaches for the diagnosis of enteric fever are not available is the unwillingness to verify inaccurate diagnoses. Physicians depend on their professional expertise to create an “accurate guess” of the origin of the disease [14]. This could lead to an overestimation of reported cases of typhoid fever in clinically suspected cases of enteric fever, since typhoid is better studied and well understood compared to paratyphoid [14]. This has been worsened by the unavailability of a vaccine against *S.* Paratyphi when compared to *S.* Typhi [15].

Since early and accurate laboratory diagnosis is a crucial phase in ensuring effective health care, reducing disease transmission, and developing potential vaccines, standard methods for *S.* Paratyphi detection are needed for the proper management of paratyphoid fever [16]. The common procedures for the detection of *S.* Paratyphi involve growing the bacteria in a culture medium, accompanied by serological, biochemical, or molecular diagnostics. However, these approaches sometimes work with poor selectivity and specificity [17]. So far, there is no true gold standard for the detection of *S.* Paratyphi. Meanwhile, more advances have been made to detect *S.* Typhi, especially in the molecular analysis field.

In this review, we highlight the current diagnostic approaches for *S.* Paratyphi, and expound on the potentials of surface plasmon resonance (SPR) as a promising alternative for the detection of *S.* Paratyphi. Although the SPR has been extensively used for bacterial detection, it is rarely used for *S.* Paratyphi, and, to the best of our knowledge, it has never been used for the clinical diagnosis of paratyphoid fever. Thus, we propose in this review, an SPR-based genoassay for the detection of *S.* Paratyphi, and suggest it as a promising future direction.

## 2. Paratyphoid Fever

Paratyphoid is caused by the bacterium *S. enterica* of the serotypes Paratyphi A, B, or C that infect the gastrointestinal tract [18]. Enteric fever was previously believed to be primarily caused by *S.* Typhi, thus, paratyphoid fever appeared to be neglected. However, emerging data from several Asian countries including India, Indonesia, Pakistan, and China show that *S*. Paratyphi is also a major cause of enteric fever [15]. Paratyphoid fever is widely spread in Asia, where the common cause is *S.* Paratyphi A, but less common in Europe and caused by *S.* Paratyphi B. On the other hand, *S.* Paratyphi C is very rare and not common in certain destinations [19,20].

Although it is known that paratyphoid fever is clinically milder than typhoid [21], approximately 5 million cases occur globally every year, with developing countries being the most affected. [20]. The symptoms of paratyphoid fever usually begin 6–30 days after exposure, and then a gradual onset of a high fever occurs over several days [22]. Headaches, loss of appetite, and weakness also commonly occur, and a skin rash with rose-colored spots manifests in some individuals. If the condition is not treated, these symptoms could last several weeks [23]. The bacteria usually spread via the consumption of food or drink contaminated with the pathogen as a result of poor hygienic practices during the preparation of food items. [24]. One of the risk factors thus includes poor sanitation, which is common among poor crowded populations and in places where there is poor hygiene [24]. It has been reported that non-domestic hygiene conditions (i.e., environmental and societal) are more relevant to paratyphoid fever, while home-related hygiene conditions are more associated with typhoid fever [25].

Sadly, the clinical diagnosis of paratyphoid fever is so far not precise [26]; it is hard to differentiate it from typhoid fever, as well as some other fever-associated diseases. Altogether, the diagnosis and monitoring of paratyphoid fever is a serious concern for clinical diagnostic experts, due to its overlapping diverse symptoms with typhoid fever. Therefore, various laboratory tests are currently being used to confirm the diagnosis of paratyphoid fever by detecting *S.* Paratyphi.

## 3. Laboratory Diagnostic Approaches

Apparently, to diagnose paratyphoid fever, most diagnostic microbiology laboratories depend on a conventional approach of culture and biochemical analysis, which often takes at least 4–5 days to obtain final results [27]. Although efforts have been made in the last three decades to establish accurate and responsive testing systems for *Salmonella*, these efforts were not focused on *S.* Paratyphi [28,29,30]. Since it is important to differentiate between *S.* Typhi and *S.* Paratyphi infections, as they cannot be differentiated clinically, microbial culture has been supported by other tests that are based on the detection of antigens, antibodies, or nucleic acid (i.e., RNA or DNA).

In addition to the bacterial culture of *S.* Paratyphi, detection and screening methods rely heavily on the Widal test, enzyme-linked immunosorbent assay (ELISA) and polymerase chain reaction (PCR) [31]. However, these methods are not suitable for large scale routine screening, because it takes a few days for the confirmation of the presence of the bacteria [28]. Moreover, the methods require highly skilled laboratory personnel to handle the equipment [28].

It is important to mention that, upon isolation of *S.* Paratyphi, it should be carefully handled, to avoid cases of laboratory-acquired infection. While it is recommended to handle the pathogen under biosafety level 3 setting, cultures and specimen should be autoclaved before final disposal [32].

### 3.1. Bacterial Culture

The most essential diagnostic approach for paratyphoid fever is the culture of *S.* Paratyphi from bone marrow, followed by other sample types [33]. Compared to blood sample, several reports have shown that culture from bone marrow specimen is more sensitive when isolating *S.* Paratyphi, and the results of bone marrow culture is rarely affected in the first few days of a patient’s usage of antibiotics [32,34]. This higher sensitivity has been attributed to the increased bacterial concentrations in bone marrow compared to other specimens [35]. In 80% to 95% of cases, the culture of bone marrow showed positive results [36].

Although a few studies have suggested using the fine-needle technique for the collection of bone marrow specimen, as it can be easily tolerated, usage of the specimen for diagnosis is uncommon in actual clinical practice [32,35]. Blood culture, a routine diagnosis preferably performed within the first 14 days of the disease, is reported to be positive in 40% to 80% of cases [33,37]. This range of sensitivity has been attributed to stage of disease, bacterial concentration in the blood, the usage of antimicrobial medicines, bacterial concentration in the broth, type of culture medium, and the period of incubation [38,39].

Although bone marrow samples yield better diagnostic results following culture of *S.* Paratyphi, other specimens, such as cerebrospinal fluid (CSF), rose spot, duodenal bile, urine, and feces, have also shown good results, despite their limitations [32]. The majority of positive CSF cultures, for instance, are from young children [32]. Furthermore, even though 70% of paratyphoid patients have shown positive rose spot culture, rose spots are considered an uncommon sign [32]. Although feces and urine are commonly used samples, it has been reported that *S. enterica* could be isolated from only about 30% of feces, and less than 1% of urine samples in patients with typhoid fever [32]. While the culture of duodenal bile samples has shown similar sensitivity as that reported for blood samples, the procedure of sample collection is difficult to tolerate, especially for young children and those with severe disease [40].

Taken together, despite current advances in diagnostic laboratories, positive culture from specimens other than the bone marrow must be carefully examined, to ensure reliability in the interpreted results [16]. Furthermore, even though blood culture is a recommended test, more than 48 h is required to obtain result [33], making rapid diagnosis and best treatment impossible.

### 3.2. Serology

Several immunoassay methods are commonly used to identify *S.* Paratyphi serologically, but they are typically costly compared to bacterial culture, and involve time-consuming and complicated sample pretreatment protocols. The immunoassay reactions to detect *Salmonella enterica* serovars are mainly focused on O and H antigens, and sometimes the Vi antigen, which is a capsular antigen present in a few *Salmonella* lineages, including *S.* Paratyphi C and *S.* Typhi [41]. Although all the serotypes of *Salmonella* harbor similar kinds of antigens, it has been reported that each serotype has a unique combination of the antigens, giving rise to distinct antigenic formula (Table 1) [11,41]. The most common serological tests that have been widely used to detect *Salmonella enterica* serovars are Widal test, ELISA, and rapid tests.

#### 3.2.1. Widal Test

**Figure 1:**
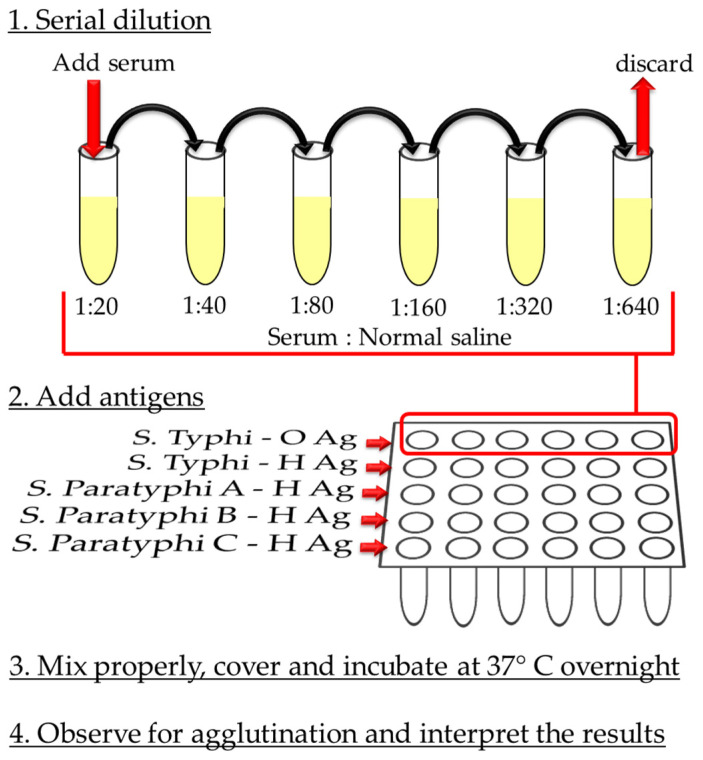
The general procedure for the Widal test tube method [44]. 1. An essential step in any Widal test procedure is the serial dilution (the number of folds could be varied). This helps to avoid false negative results, due to Prozone phenomenon (high antibody (Ab) titer compared to the number of antigens (Ag)); 2. Reagents containing specific *S. enterica* serovars antigens are added. Although the test relies on the antigenic structure of each serovar (Table 1), the O somatic Ag of *S.* Paratyphi is not used, because of the factor 12 that is also present in the O Ag of *S.* Typhi; 3. Upon addition of Ags, the setup is properly mixed and then incubated. Usually, the duration of incubation is up to 18 h at 37 °C; 4. After incubation, the sample is vortexed and then agglutination is viewed by the naked eye if the result is positive. The final result is the highest dilution (titer) with a visible agglutination.

In 1896, Georges Fernand Widal developed a serological test to detect the H and O antigen-based agglutination in the serum of patients suspected to be infected with *Salmonella enterica* [42]. This test became known as the Widal test, and it is a conventional test to differentiate between typhoid and paratyphoid fever [33]. The core concept of the Widal test is that the antigens (O and H) are present in the reagent that is added to the patient’s serum, which contains antibodies that conjugate to the respective antigens and give visible agglutinates [43]. Currently, the Widal test is known to be either a qualitative detection test if the slide agglutination method is used, or semi-quantitative in the case of the tube method (Figure 1) [44].

For over a century, the Widal test has been an invaluable tool for the detection of *S.* Paratyphi and *S.* Typhi. However, modern technological advancements have revealed some limitations of the procedure. If a sample is from an unvaccinated individual who has never been infected, the test result could be considered significantly correct. However, the result may have no diagnostic significance if the patient has been previously infected or vaccinated [45]. In addition, it has been suggested that some pathogens may present antigenic determinants, similar to those of *Salmonella*, thereby further confounding the reliability and interpretation of Widal test results [45]. Therefore, increased attempts have been made to invent a more sensitive, rapid, accurate, and reliable method for the diagnosis of paratyphoid and typhoid fever.

#### 3.2.2. ELISA

The most common immunochemical detection assay for *Salmonella* is ELISA. The assay has an efficient detection limit that starts approximately from 10^4^ to 10^7^ colony-forming unit (CFU) per mL (CFU/mL) [46,47,48]. As antibodies are critical factors in any immunoassay-based detection method, ELISA also relies on generated Abs to either capture the vital antigens (especially O and H antigens) or to help in the detection of serum Abs against those main surface antigens [49,50].

Like some other pathogens, the major types of ELISA used to detect *S.* Paratyphi include indirect, sandwich, and competitive ELISAs [51]. The indirect ELISA is based on the use of specific antigen-coated wells to detect the representative antibodies from the specimen. This is followed by the addition of a formulated enzyme-conjugated secondary antibodies, which improves the rate of detection [51,52]. The indirect ELISA has been used to detect immunoglobulin G (IgG) and IgM to O antigen [53]. Sandwich ELISA, on the other hand, is based on the use of formulated antibody-coated wells to detect representative antigens, followed by the addition of formulated enzyme-conjugated secondary antibodies [51]. Several studies have used sandwich ELISA with capture antibodies and H or O monoclonal antibodies (mAbs) as a detection antibody [50,54,55]. The competitive ELISA is based on preliminary incubation of antibodies with the antigens to be measured, followed by the addition of the antibody-antigen mixture to antigen-coated wells, and a subsequent addition of formulated enzyme-conjugated secondary antibodies [51].

Ayyildiz and colleagues conducted a study using 168 serum samples to determine the titer of antibodies against the pathogens of paratyphoid and typhoid fever using Widal test and ELISA [56]. This study showed lower nonspecific reactions and approximately four to six times higher titer in ELISA, as compared to the Widal test [56]. Additionally, a study of an *S.* Paratyphi A outbreak in China was conducted, using ELISA to measure the levels of serum IgG against the H antigen [55].

In other studies on *S.* Paratyphi A in patients, ELISA has also been used to detect antibodies against gene products of the outer membrane protein X (*OmpX*), *SpaO*, *H1a*, and *PagC* genes [57,58,59]. It was suggested that the gene products could be reliable target antigens in cases of *S.* Paratyphi A infections [57,58,59]. Similarly, the Vi antigen has been targeted and utilized in ELISA-based diagnosis of chronic carriers since antibodies to the Vi antigen tend to appear late in the course of infection (making it less useful for the diagnosis of acute infection with *S.* Paratyphi) [60,61]. However, it is important to note that both *S.* Typhi and *S.* Paratyphi C share this factor.

Although ELISA has been shown to provide superior results compared to the Widal test against the same antigens, it is reported to have limitations of specificity, as observed in the Widal test [60]. Altogether, although various types of ELISA have been used in the past few decades for the detection of *S.* Paratyphi [62,63], the technique still requires further developments to improve its diagnostic accuracy for paratyphoid fever.

#### 3.2.3. Other Tests

Other serological diagnostic tests have also been developed and used for *S.* Paratyphi detection. A notable example is the typhoid and paratyphoid test (TPTest), which has efficiently been used to detect specific IgA to *Salmonella* in patients’ samples within 24 h [64]. The test showed flexibility in differentiating between current and past infection. Compared to blood culture, the TPTest showed 100% sensitivity and 78–97% specificity, based on predefined true negative samples [64]. Therefore, the TPTest can be considered a relatively rapid and reliable diagnostic assay, and could be very useful, particularly in endemic regions. However, the assay still requires further evaluation to support its usage [64].

Rapid diagnostic tests (RDTs) have also been employed for the detection of *S.* Paratyphi. RDTs are based on various principles, including agglutination, lateral flow, solid-phase, and flow-through methods that can detect antigens or antibodies [65]. Additionally, there are many types and forms of RDTs that allow the use of serum, whole blood, or urine samples [65]. In a comparative meta-analytical study on accuracy of diagnostic tests for pediatric population with paratyphoid and typhoid fever, the lateral-flow IgG immunoassay (rapid test), TPTest, and the reverse passive hemagglutination (RPH) assay, showed a better diagnostic accuracy, compared to ELISA and Widal tests [66]. Another study compared several *Salmonella* diagnostic tests, and reported the TPTest to be the most sensitive and specific, outperforming blood culture, the Widal, test and commercially available RDTs (Typhidot and Tubex) [67]. Despite some of the recorded successes in the use of these other serological tests, a more robust evaluation needs to be conducted to guarantee their reliability, especially when using human serum, given the polyvalent nature of some antigens.

### 3.3. Nucleic Acid-Based Diagnostics

As the CFU/mL of blood is often low, especially during antimicrobial therapy, the volume of blood collected for serology or culture tests is critical, as it may lead to an inability to isolate the bacteria [41]. The emergence of nucleic acid-based diagnostics has greatly remedied the concerns of limited quantity of bacteria in blood and other samples [16]. However, this diagnostic approach requires a proper understanding of a pathogen’s genetics.

It has been shown that *S.* Paratyphi shares similar genetic content to that of *S.* Typhi, but with a possibility to have more recent evolutionary origins [68]. Furthermore, 1% to 3% of the gene content of *S.* Paratyphi A and *S.* Typhi are unique [60]. However, the functions of some *S.* Paratyphi genes are yet to be fully known, underscoring the need for further investigations to reveal potential targets for *S.* Paratyphi nucleic acid-based diagnostics.

PCR, a technique which allows the amplification of genetic material [69], has been used for the detection of bacteria from a wide range of biological samples (including clinical and non-clinical) [70,71]. The method typically involves the extraction of genetic material (DNA or RNA), followed by an amplification phase (using a thermal cycler) to generate multiple copies of a genomic region of interest which could then be further studied [72,73]. Given its high sensitivity and specificity, PCR is an indispensable tool in the laboratory diagnosis of infectious diseases [74]. In addition, the method is relatively rapid, and its results are unaffected by sample volume, antibiotics consumption, vaccination status, and the stage of infection [74].

Several genes, including the O antigen genes, H antigen genes, Vi capsular antigen gene and 16S rRNA gene, have been targeted to detect and study *S. enterica* [27]. The *cytolysin A* (*ClyA*, *HlyE* or *SheA*) gene, which was earlier discovered in *E. coli*, has also been reported to be contained in *S*. Paratyphi A and *S*. Typhi, but not in other *Salmonella* serovars [75,76,77]. Tennant and colleagues developed specific probes and primers for the *ClyA* gene, and found them effective in detecting *S*. Paratyphi A and *S*. Typhi in blood using the quantitative real-time PCR (qPCR) [31]. Similarly, another study that developed a multiplex PCR (mPCR) targeted the *outer membrane protein C* (*ompC*) gene, to detect *S*. Paratyphi A and *S*. Typhi. In the study, a sensitivity of 4.5 × 10^4^ CFU/mL was reported [27]. However, the *fliC-a* gene, which is specific to S. Paratyphi A [78], as well as the intergenic region (SSPAI) between genes *SSPA1723a* and *SSPA1724* [27], have been targeted to distinguish *S*. Paratyphi A from other serovars.

Despite the distinctive characteristics of PCR as a relatively rapid and sensitive diagnostic tool, it suffers limitations, such as high cost, high risk of contamination that could yield false results, and the need for highly skilled operators [71,79,80].

### 3.4. SPR: A Promising Technology for Paratyphoid Diagnosis

Biosensors provide hopeful options for bacterial detection with high sensitivity and specificity [81]. SPR is one of the optical biosensor technologies based on an interaction of an evanescent wave field with experimental analyte(s) on a sensor surface [82]. The analytes can be DNAs, RNAs, proteins, antibodies, and antigens, which are tightly bound with selective immobilized ligands on the surface sensor [83]. Various strategies for surface sensor preparation to immobilize ligands have been used, as shown in Table 2.

Prior to the actual SPR detection, clinical samples collected from infected subjects are processed to isolate and culture the pathogen in order to obtain the required concentration (Section 3.1), while antigens and antibodies can be directly assessed from the samples [88]. For DNA/RNA detection, there is a need for the extraction of nucleic acid from the cultured pathogen, using a commercially available kit, followed by PCR before the sensing [93].

**Figure 2:**
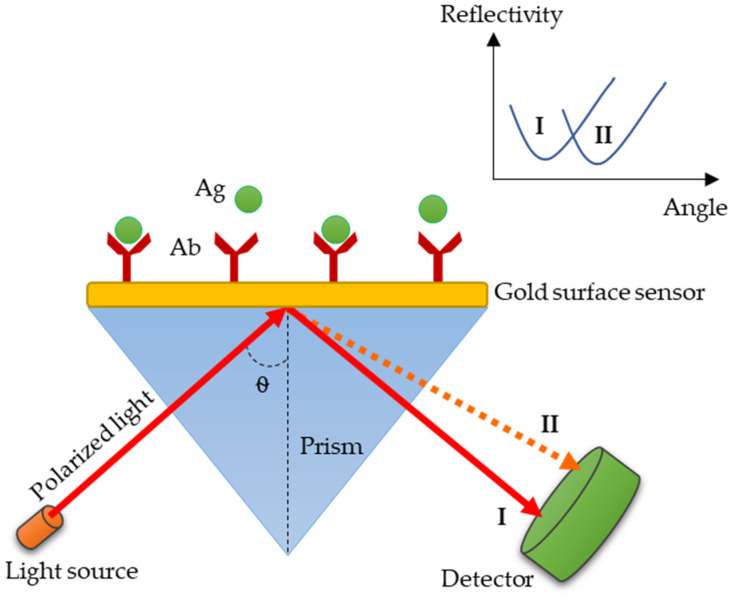
A schematic representation of surface plasmon resonance (SPR) biosensor based on antibody/antigen binding. This SPR system is commonly used for bacterial detection. Before the bindings occur, the angle of reflection is at I, and after the analyte (Ag) binds to the immobilized ligand (Ab), the reflection angle widens, due to the increase in refractive indices on the gold surface [96].

The phenomena of an evanescent wave in SPR is created when a polarized light experiences reflection on a metal-based surface (known as surface sensor) incorporated with a prism in sensing systems [94]. Changes in density (concentration) of the analyte bring relative changes to its refractive index (RI), which then leads to the alteration of the angle of the light reflection on the surface sensor [94]. Gold (Au) has been characterized as the best metal for surface sensors, especially with the biological samples, due to its oxidation stability (inert) compared to other suitable metals, such as silver, aluminum, and copper [95]. At a receptor side (detector), a measurable output in terms of binding affinity, binding kinetics, as well as changes of reflection light angle for the determination of the system’s sensitivity, can be obtained (Figure 2).

**Figure 3:**
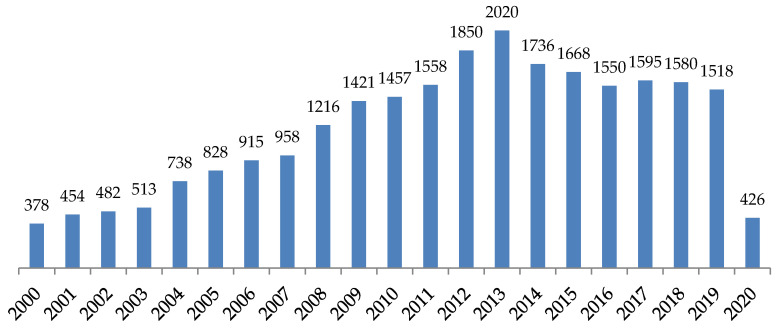
Annual publications on the SPR-based methods in the last two decades, according to PubMed databases (as at 20 March 2020).

SPR was proposed for the first time in 1983 by Liedberg and his team [97]. This proposed SPR method was primarily for gas detection. However, the first medical application of SPR in pathogens detection was in 1998 by Fratamico et al., who successfully utilized it to get sensitive, rapid, and quantitative results [98]. During the last two decades, SPR has continuously attracted much interest in various research areas (including bacterial detection), as shown in Figure 3. Several biomolecules, including cells, nucleic acids, antibodies, antigens, proteins, and carbohydrates, have been successfully detected by SPR-methods [94,99]. This has opened the door for the utilization of SPR as a sensitive detector for bacteria in real-time in areas such as pharmaceutics, clinical diagnostics, food analysis, and environmental monitoring [94,99].

The efficiency of any SPR-based detection relies mainly on analyte/ligand interaction, as well as ligand/surface-sensor binding. While the ligands used for immobilization can differ, the concepts of SPR-based methods are similar [100,101]. As the binding of the desired ligand with the analyte is a key step in SPR development, several approaches, including antibody/antigen and protein/protein interactions, have been tested [102]. Antibodies have been the most extensively used ligands in SPR for bacterial detection, and are commonly known as SPR-based immunoassay or SPR immunosensors, as shown in Table 3. The SPR-based immunoassays have been fabricated for bacterial detection by three main detection formats similar to ELISA; direct, sandwich, and competitive detections [103]. However, most studies have used SPR-based detection methods for environmental monitoring and food analysis, and not for clinical diagnostics (Table 3).

For the serotyping of *Salmonella*, the targets are similar to those in serological tests; the O and H surface antigens can be captured on SPR by immobilized antibodies [104]. Mainly, the SPR-based sandwich immunoassay is most commonly used for detection of *Salmonella*; where anti-*Salmonella* mAbs or polyclonal Abs (pAb) are fixed on the sensor surface to capture various *Salmonella* serovars, and then specific antibodies for O or H antigens helps to detect each serovar [105]. However, among several studies that utilized SPR-based methods for bacteria detection, only two studies targeted *S.* Paratyphi (Table 3); Perçin et al. [106] detected *S.* Paratyphi isolated from bacterial culture using a novel SPR method that was based on a special microcontact imprinted sensor chip, and demonstrated 2.5 × 10^6^ CFU/mL as the lowest detected concentration, while Oh et al. [107] detected *S.* Paratyphi isolated from bacterial culture using SPR-based immunoassay, with 10^2^ CFU/mL as the lowest detected concentration.

Although the SPR-based methods can allow whole cell detection of *Salmonella* even from crude samples and without purification, some limitations have also been assumed. For instance, Fu et al. pointed that bacteria (∼2 μm long) has a rigid body bigger than the Au NPs (∼30 nm), which could limit the contact area between them, and consequently, affect the expected change in the surrounding environment, and the resultant plasmon peak shift [108]. However, the recent emergence of SPR-based genoassay (genosensor) has allowed the detection of bacteria based on immobilizing specific DNA probes, without a need for whole-cell detection [109]. Interestingly, SPR-based genoassay overcomes even the limitations in PCR methods, as it can directly test extracted DNA (i.e., without amplification) and give highly sensitive results [109].

An SPR-based genoassay has been performed to detect *S.* Typhi based on thiolated self-assembled monolayer of single-stranded DNA (ssDNA) as a DNA probe, which is designed for the Vi-antigen gene of *S.* Typhi [130]. In this study, specific ssDNA probes were immobilized onto gold film, and the assay records the lowest detection concentration (0.019 ng/mL^−1^), when compared to several other SPR-based bacterial detection studies (Table 3). Upon using the cell systematic evolution of ligands by exponential enrichment (Cell-SELEX) technique in a study, ssDNA probes that could detect *S.* Enteritidis and *S.* Typhimurium were identified [140]. The study recommended using ssDNA probes for ultra-sensitive and specific detection of *Salmonella* with fabricated genosensors [140]. To our knowledge, no study, to date, has developed and used genosensor for the detection of *S.* Paratyphi. With the advantages and promise of SPR-based genoassays, it would be pertinent to explore them in the clinical diagnosis of paratyphoid fever.

From a diagnostic perspective, one of the most crucial parameter in any SPR sensor is the limit of detection (LoD), as it indicates the effectiveness of SPR. Usually, it is obtained indirectly from a linear calibration curve—a curve which consist of a linear regression of a set of measurements derived from response of the SPR instrument versus analyte concentration [141]. The LoD presents the least concentration which can give an output signal, and it is equal to three times the system noise (standard deviation (*σ*)) divided by the sensitivity (*S*) (3*σ*/*S*) [142]. The value of *S* can be in any terms, including RI, concentration, or molar [143]. The LoD depends on the features of target-probe molecules (i.e., molecular weight, binding affinity, and optical property), in addition to the surface coverage of the probe molecule [83]. Although it is well-known that SPR-based methods can detect at low LoD, there is still a continuous competition towards developing these methods to be more user-friendly with even lower LoD and higher accuracy.

## 4. Conclusions and Future Directions

The emergence of paratyphoid fever worldwide has led to increase in commercially available immunoassays for early diagnosis, but comes with recognized limitations. Most conventional methods to detect *S*. Paratyphi after the infection are highly dependent on lab testing, and would usually take a few days for confirmation of the presence of the bacteria. Hence, there is a need for a rapid, low-cost, simple diagnostic method with high sensitivity and specificity to be used as a detection tool for *S*. Paratyphi. Fortunately, SPR offers those attributes [144]. The development of an optimized SPR-based genoassay to detect S. Paratyphi will be crucial for the rapid, real-time, and label-free detection and efficient diagnosis of paratyphoid fever.

**Figure 4:**
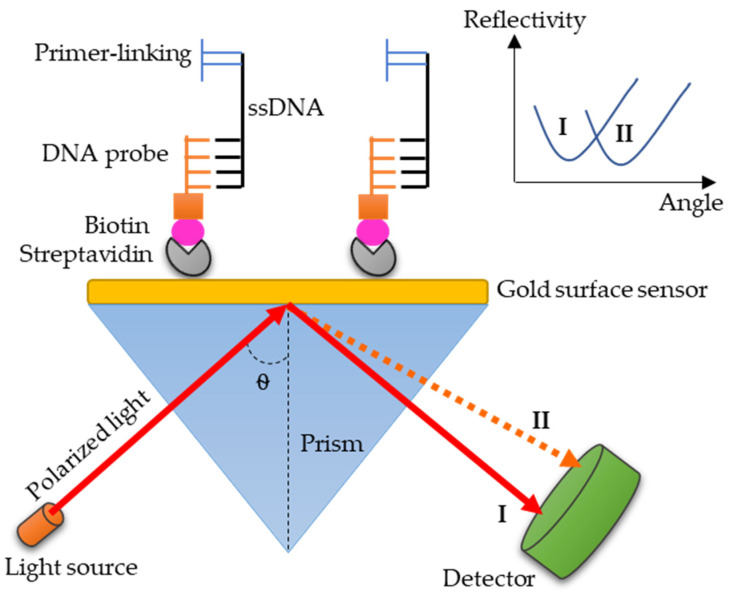
A proposed SPR-based genoassay to detect *S*. Paratyphi. Immobilization of biotinylated DNA probe onto the gold surface can be done, based on the cross-linking (capturing) method. Streptavidin will be functionalized with biotin (coupled with DNA probe) as a protein capture agent. The binding of the immobilized biotinylated DNA probe to ssDNA will produce a specific change in the light output reflected from the gold-based surface. By monitoring both the immobilization process, as well as the reaction between the biotinylated DNA probe and ssDNA, the changes in refractive index, absorbance, reflectance, kinetic, binding assay, and spectrum can be obtained and analyzed, allowing the determination of the most sensitive parameter within this approach.

The potential of the SPR for *Salmonella* (live, dead, protein or DNA) detection, including *S.* Typhi and *S.* Typhimurium, has been widely studied [126,145,146], but there are no studies on SPR to detect the DNA of *S.* Paratyphi using specific DNA probes. Figure 4 illustrates a proposed SPR-based genoassay to detect *S.* Paratyphi, which can be explored in future studies. The immobilization of a biotinylated DNA probe onto a layer of streptavidin in SPR-based optical sensing provides a stable immobilization structure and prevents the DNA probe from slipping off during the regeneration process. The biotinylated DNA probe would be strongly attached to ssDNA due to its specificity. Note that the presence of primer-linking attached to the other end of ssDNA is to avoid it from binding to other ssDNA in SPR, in order to prevent the formation of double-stranded DNA (dsDNA) [93]. The different number of bindings between DNA probe and ssDNA would vary the refractive indices on the surface sensor, and thus lead to changes in reflection angle from I to II, as depicted in Figure 4.

One of the challenges that might be encountered in the proposed SPR-based genoassay (Figure 4) is the design of a highly specific DNA probe to ensure an accurate result. Another challenge would be in the regeneration process, where the reusable biotinylated DNA probe could easily be inactivated or detached from the surface sensor, which would be for various reasons, such as the use of unsuitable regeneration solutions, incorrect regeneration time set up, unstable structure, or short lifespan of the DNA probe itself. With an unusable or unrecyclable DNA probe, the cost of operation would increase. When integrating gold-based sensors, the interactions between ligand/probe must be clearly understood to hasten the development of a reliable SPR-based diagnostic tool for the diagnosis of paratyphoid fever in the near future.

With the drive of digital transformation through technological advancement and convergence towards fifth generation (5G) technology, the technology of SPR is driven by the technological drivers in internet of things (IoT) categories. The interconnection between computing devices and databases via the internet could be harnessed and applied to SPR, so that data or information can be rapidly and efficiently transferred to health staff for prompt decision. In the future, a developed SPR device could be equipped with DNA/RNA extraction kit coupled with mobile PCR equipment, so that in-situ lab-free measurements can be successfully achieved [147]. This will ultimately spur future development of a commercial optical sensor, which utilizes the SPR method and could be incorporated to a computing device for the early detection of *S.* Paratyphi.

## Figures and Tables

**Table 1:** Antigenic signatures that have been utilized in *Salmonella enterica* serological and molecular detection tests [11,41].

Antigen Name	Antigenic Virulence Factors			
	*S.* Paratyphi A	*S.* Paratyphi B	*S.* Paratyphi C	*S.* Typhi
H flagellar	a, 1, 5	b, 1, 2	c, 1, 5	d
O somatic	1, 2, 12	1, 4, 5, 12	6, 7	9, 12
Capsular antigen	-	-	Vi	Vi

**Table 2:** Surface modification methods for ligand immobilization.

Method	Advantage(s)	Disadvantage(s)	Reference(s)
Physical adsorption	For study of membrane-associated protein	The immobilized ligands are formed in random oriented order	[84,85]
Thiol-based	Covalent binding; thus, provides strong immobilization of ligand with thiol group and in homogenous orientation	Chemical synthesis and protein engineering need to be carried out if thiol group is lacking	[86,87]
Self-assembled monolayer based	Covalent binding; thus, provides strong immobilization of ligand with amine-coupling group and in homogenous orientation. This method is the simplest	Efficiency of immobilization can be decreased due to non-specific biding of ligand onto the surface	[88]
Capture	This method is used when the covalent immobilization process is not sufficient enough. Common techniques: streptavidin-biotin and antibody-antigen	Both analyte and ligand are removed during regeneration, so a new ligand is required, thus, increasing cost	[89,90]
Polymer film deposition	Provides high sensitivity	Weak binding to the sensor chip through non-covalent forces	[91,92]

**Table 3:** Previous studies that have utilized SPR for bacterial detection.

Study ID [Reference]	Detected Bacteria	Sample	Principle of Immobilization	Limit of Detection
Arya 2011 [110]	*Escherichia coli* K12	Bacterial culture	T4-based bioassay	7 × 10^2^ CFU/mL^−1^
Aura 2017 [109]	*Staphylococcal enterotoxin A*, *Staphylococcus aureus* and *Listeria monocytogenes*	Milk	Ab/Ag immunoassay and PNA/SSO probes-based genoassay	0.05 µg/mL
Bhandari 2019 [111]	*S.* Typhimurium	Romaine lettuce	Ab/Ag immunoassay	0.9 log CFU/g
Bhandari 2019 [112]	*S.* Typhimurium	Bacterial culture	Ab/Ag immunoassay	-
Barlen 2007 [105]	*S.* Typhimurium and *S.* Enteritidis	Milk	Ab/Ag immunoassay	2.50 × 10^5^ cells/mL^−1^
Barlen 2009 [113]	*S.* Enteritidis (antibodies)	Bacterial culture	Ab/Ag immunoassay	10^10^ cells/mL^−1^
Bokken 2003 [114]	*Salmonella* group B, D and E	Bacterial culture	Ab/Ag immunoassay	10^7^ CFU/mL^−1^
Chen 2017 [115]	*S.* Enteritidis, *S.* Kentucky, *S.* Infantis, *S.* Javiana, *S.* Heidelberg and *S.* Typhimurium	Chicken carcass	Ab/Ag immunoassay	2.1 × 10^6^ CFU/mL
Eser 2015 [116]	*S.* Enteritidis	Bacterial culture	Ab/Ag immunoassay	10^2^ CFU/mL
Fratamico 1998 [98]	*E. coli* O157:H7	Bacterial culture	Ab/Ag immunoassay	10^7^ CFU/mL
Fu 2009 [108]	*S.* Typhimurium	Bacterial culture	Ab/Ag immunoassay	10^4^ CFU/mL
Jongerius 2002 [117]	*S.* Enteritidis and *S.* Typhimurium (antibodies)	Serum from infected chickens	Ab/Ag immunoassay	-
Jyoung 2006 [118]	*Vibrio cholerae* O1	Bacterial culture	Ab/Ag immunoassay	10^5^ cells/mL
Kim 2006 [119]	*Mycoplasma hyopneumoniae* (antibodies)	Serum from infected pigs	Ab/Ag immunoassay	-
Koubova 2001 [120]	*S.* Typhimurium and *L. monocytogenes*	Bacterial culture	Ab/Ag immunoassay	10^6^ cells/mL
Lan 2008 [121]	*S.* Typhimurium	Chicken carcass	Ab/Ag immunoassay	10^6^ CFU/mL
Lukose 2018 [122]	*S.* Typhimurium	Bacterial culture	Ab/Ag immunoassay	10^6^ CFU/mL^−1^
Mazumdar 2007 [123]	*S.* Typhimurium	Milk	Ab/Ag immunoassay	1.25 × 10^5^ cells/mL^−1^
Mazumdar 2008 [124]	*S.* Typhimurium (antibodies)	Serum from infected pigs	Ab/Ag immunoassay	67.5 µg/mL^−1^
Mazumdar 2010 [104]	*Salmonella* group B, C and D	Bacterial culture	Ab/Ag immunoassay	10^10^ cells/mL^−1^
Meeusen 2005 [125]	*E. coli* O157:H7	Bacterial culture	Ab/Ag immunoassay	8.7 × 10^6^ CFU/mL
Nguyena 2016 [126]	*S.* Typhimurium	Bacterial culture	Ab/Ag immunoassay	10^7^ CFU/mL
Oh 2004 [107]	*S.* Paratyphi	Bacterial culture	Ab/Ag immunoassay	10^2^ CFU/mL
Oh 2005 [127]	*E. coli* O157:H7, *S.* Typhimurium, *Legionella pneumophila* and *Yersinia enterocolitica*	Bacterial culture	Ab/Ag immunoassay	10^5^ CFU/mL
Perçin 2017 [106]	*S.* Paratyphi	Bacterial culture	A special microcontact imprinted sensor chip programed to detect *S.* Paratyphi	2.5 × 10^6^ CFU/mL
Perkins 2000 [128]	*Bacillus subtilis* (spore)	Bacterial culture	Ab/Ag immunoassay	10^7^ mL^−1^
Si 2001 [129]	*S.* Enteritidis	Bacterial culture	Ab/Ag immunoassay	10^5^ cells/mL
Singh 2014 [130]	*S.* Typhi	ssDNA extracted from bacterial culture	DNA self-assembly	0.019 µg/mL^−1^
Subramanian 2006 [131]	*E. coli* O157:H7	Bacterial culture	Ab/Ag immunoassay	10^4^ CFU/mL
Subramanian 2006 [132]	*E. coli* O157:H7	Apple juice	Ab/Ag immunoassay	10^6^ CFU/mL
Subramanian 2006 [133]	*S. aureus*	Bacterial culture	Ab/Ag immunoassay	10^5^ CFU/mL
Taheri 2016 [134]	*V. cholerae* O1 serovar Ogawa	Bacterial culture	Ab/Ag immunoassay	43 cells/mL
Thomas 2006 [135]	*S.* Enteritidis (antibodies)	Eggs from chickens infected with *Salmonella enteritidis*	Ab/Ag immunoassay	-
Usachev 2014 [136]	*E. coli* K12	Bacterial culture	Ab/Ag immunoassay	1.5 × 10^3^ CFU/mL^−1^
Waswa 2006 [137]	*S.* Enteritidis, *E. coli* O26, K12, NM and H16	Milk	Ab/Ag immunoassay	23 CFU/mL
Waswa 2007 [138]	*E. coli* O157:H7	Milk, apple juice and ground beef	Ab/Ag immunoassay	10^2^ CFU/mL
Zhang 2017 [139]	*E. coli* O157:H7, *S.* Enteritidis and *L. monocytogenes*	Bacterial culture	Ab/Ag immunoassay	6 CFU/25 g

CFU, colony-forming unit; mL, milliliter; µg, microgram; Ab, antibody; Ag, antigen; ssDNA, single-stranded DNA; PNA, peptide nucleic acid; SSO, sequence-specific oligonucleotide; T4, T4-bacteriophage.
